# Combining BN-PAGE and microscopy techniques to investigate pigment-protein complexes and plastid transitions in citrus fruit

**DOI:** 10.1186/s13007-022-00956-1

**Published:** 2022-11-19

**Authors:** Jinli Gong, Hang Zhang, Yunliu Zeng, Yunjiang Cheng, Xuepeng Sun, Pengwei Wang

**Affiliations:** 1 Collaborative Innovation Center for Efficient and Green Production of Agriculture in Mountainous Areas of Zhejiang Province, College of Horticulture Science, Zhejiang A&F University, Hangzhou, 311300 Zhejiang China; 2 Key Laboratory of Horticultural Plant Biology (Ministry of Education), College of Horticulture and Forestry Science, Huazhong Agricultural University, Wuhan, 430070 Hubei China; 3 National R&D Centre for Citrus Preservation, Huazhong Agricultural University, Wuhan, 430070 Hubei China; 4 Hubei Hongshan Laboratory, Wuhan, 430070 China

**Keywords:** Plastid, Pigment-protein complex, Chlorophyll, Carotenoid, BN-PAGE, Citrus fruit

## Abstract

**Background:**

Chlorophyll and carotenoids, the most widely distributed lipophilic pigments in plants, contribute to fruit coloration during development and ripening. These pigments are assembled with pigment-protein complexes localized at plastid membrane. Pigment-protein complexes are essential for multiple cellular processes, however, their identity and composition in fruit have yet to be characterized.

**Results:**

By using BN-PAGE technique in combination with microscopy, we studied pigment-protein complexes and plastid transformation in the purified plastids from the exocarp of citrus fruit. The discontinuous sucrose gradient centrifugation was used to isolate total plastids from kumquat fruit, and the purity of isolated plastids was assessed by microscopy observation and western blot analysis. The isolated plastids at different coloring stages were subjected to pigment autofluorescence observation, western blot, two-dimensional electrophoresis analysis and BN-PAGE assessment. Our results demonstrated that (i) chloroplasts differentiate into chromoplasts during fruit coloring, and this differentiation is accompanied with a decrease in the chlorophyll/carotenoid ratio; (ii) BN-PAGE analysis reveals the profiles of macromolecular protein complexes among different types of plastids in citrus fruit; and (iii) the degradation rate of chlorophyll-protein complexes varies during the transition from chloroplasts to chromoplasts, with the stability generally following the order of LHCII > PS II core > LHC I > PS I core.

**Conclusions:**

Our optimized methods for both plastid separation and BN-PAGE assessment provide an opportunity for developing a better understanding of pigment-protein complexes and plastid transitions in plant fruit. These attempts also have the potential for expanding our knowledge on the sub-cellular level synchronism of protein changes and pigment metabolism during the transition from chloroplasts to chromoplasts.

**Supplementary Information:**

The online version contains supplementary material available at 10.1186/s13007-022-00956-1.

## Background

Plastids are a unique family of organelles in plant cells including chloroplasts, which carry chlorophyll for photosynthesis in green tissues (e.g., leaf and green fruit), and chromoplasts, which are a type of non-photosynthetic plastids responsible for carotenoid synthesis and storage in specific tissues, such as flowers, fruit and occasionally roots and leaves. Carotenoids are synthesized in chloroplasts at the early developmental stages of fruit, and accumulate in chromoplasts at the later stages, leading to distinctive colors of fruit that is critical for visual attraction and the beneficial effect on health [1]. In many fruit such as citrus fruit, transition of color from green to red/yellow is commonly attributed to the differentiation of chloroplasts into chromoplasts, which is distinguished by decomposition of photosynthetic apparatus and a dramatic increase in carotenoid accumulation [2]. In citrus, chromoplasts are generated through conversion of amyloplasts in the flesh [3], and from chloroplasts in the pericarp during fruit maturation [4].

Chloroplasts are membrane-bound organelles [5], which consist of phospholipid bilayers where proteins associated with photosynthesis and metabolic pathways are embedded [6]. In addition to the envelope, chloroplasts also possess internal membrane bilayers called thylakoids. The energy-generating capability of thylakoid membranes is dependent on the action of four different supramolecular protein complexes, including photosystem (PS) I, PS II, cytochrome b_6_f complex, and ATP synthase [7]. Many proteins, including those involved in the electron transport chain, and photosynthetic pigments (i.e. chlorophyll, xanthophylls, carotenoids and phycobilins) are assembled into pigment-protein complexes and embedded within the granum membrane. The photosynthetic pigment-protein complex is an important component of light collection apparatus in plants, which effectively transmits absorbed light energy to the reaction center for storage [8]. Carotenoid-protein complexes can modulate photosynthetic efficiency by participating in non-photochemical quenching to dissipate excess excitation energy, which is important for protection of plastids from photo-oxidative damage [9] due to that conversion of chloroplasts to chromoplasts occurs along with aging [10]. However, differentiation of chloroplasts to chromoplasts is a very rapid process, therefore, the abundance of supramolecular protein complexes in chromoplasts is low. As a result, little is known about the intermediate plastids and changes of pigment-protein complexes during fruit coloring process.

Citrus is one of the most important fruit crops in the world, however, some citrus cultivars undergo uneven degreening during ripening and postharvest storage even after they reach maturity [11], which is attributed to abnormal or delayed conversion of chloroplasts to chromoplasts and chlorophylls to carotenoids. It is particularly important for citrus fruit as postharvest degreening is an important marketing attribute that affects the overall quality and price [12]. Many previous studies have attempted to use high-throughput omics techniques to analyze protein and metabolic changes during coloring of citrus fruit [13–18]. However, due to genetic and morphological complexity, it is difficult to identify plastid protein complexes that are important for coloration of citrus fruit. Isolation of plastids is a prerequisite for the in-depth study of plastid function during citrus fruit ripening. Although there are studies for plastid isolation in limited plant species, including those using sucrose gradient to isolate chromoplasts and suborganellar compartments in different vegetables [19, 20] and that using Nycodenz density gradient for plastid isolation in tomato [21] and sweet orange pulp [18], isolation of chloroplasts or chromoplasts from the peel of citrus fruit is still very challenging due to the presence of large amounts of essential oils and acidic juices in the fruit. In this work, we identified a citrus variety, the ‘Hua Pi’ kumquat (*Fortunella crassifolia* Swingle), whose oil-free property greatly reduces the difficulty of plastid isolation from citrus peel. The intact plastids were purified by density gradient centrifugation, and laser scanning confocal microscopy was employed to monitor pigment fluorescence of plastids isolated at different developmental stages. Besides, an optimized blue native (BN)-PAGE method was introduced to separate membrane protein complexes from purified plastids. Overall, this study provides an important perspective and research method for understanding the mechanism of chromoplast differentiation and identification of multiprotein complexes in fruit.

## Materials and methods

### Plant materials

‘Hua Pi’ kumquat fruit (*Fortunella crassifolia* Swingle) was collected from Guangxi Academy of Specialty Crops (Guilin, Guangxi, China), and the outer peel was used for plastid isolation at 140 days (green), 170 days (breaker), and 200 days (orange) after flowering, respectively.

### Measurement of total chlorophylls and carotenoids

Total chlorophyll was determined as described by Wellburn [22]. Samples were ground into powder, and approximately 0.2 g of powder was added to 10 mL pre-cooled chlorophyll extraction buffer (acetone: ethanol = 2:1). After 24 h of shaking at 4 °C in the dark, the leaching liquor was centrifuged at 5000 r/min for 10 min. The supernatant was estimated by measuring the optical density at 663 nm and 645 nm with the Infinite200 Pro microplate reader (Tecan). Total chlorophyll was calculated with the equation: total chlorophyll (mg/g FW) = (8.04 A_663_ + 20.2 A_645_) × volume (L)/fresh weight (g).

Total carotenoids were extracted using the extracting solvent (hexane: acetone: ethanol = 50: 25: 25, v/v) by ultrasonic vibration under dark conditions at 4 °C. After several repeated extractions until the supernatant became colorless, the supernatant was mixed and diluted to 25 mL with hexane. The absorbance was determined at the wavelength of 450 nm using a spectrophotometer (U-2000; Hitachi, Japan) and the total carotenoids were estimated using the extinction coefficient of β-carotene, *E*^1%^ = 2505 [23].

### Isolation and purification of plastids

**Fig. 1:**
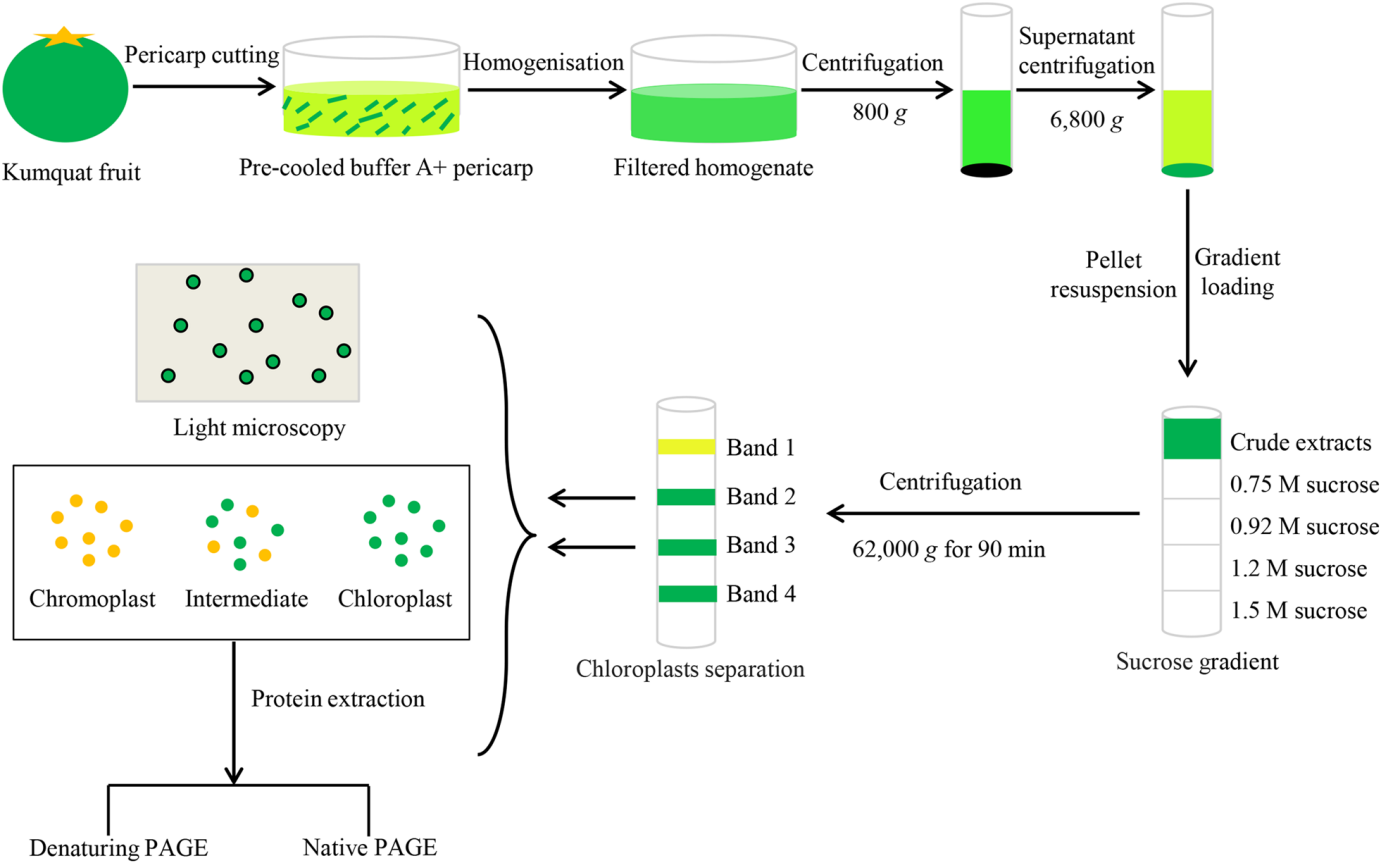
Workflow of plastid isolation and purification from the peel of kumquat fruit using sucrose density gradient centrifugation

Plastids were isolated by using sucrose density gradient centrifugation (Fig. 1) according to the method described by Barsan et al. [20] with some modifications. All materials and solutions were kept at 2–4 °C during plastid isolation and purification. Fruit was washed with distilled water. After drying, approximately 300 g of pericarp tissue was cut into small pieces with a razor blade and mixed in 500 mL pre-cooled buffer A (50 mM Tricine-NaOH pH 7.8, 5 mM KCl, 0.3 M sucrose, 0.5% (w/v) soluble polyvinylpyrrolidone, and 0.1% (w/v) bovine serum albumin). The pericarp tissues in buffer A were poured in the jar of Joyoung blender precooled at 4 °C. Blending should be performed gently by three or four short pulses (no more than 15 s each) at minimum speed to homogenize the tissues. The homogenized sample was first filtered through eight layers of gauze and then through two layers of miracloth (Calbiochem). The pooled filtrate was centrifuged at 800 × g for 5 min. The supernatant was recovered and centrifuged at 6,800 × g for 10 min. The obtained pellet was carefully re-suspended in 20 mL buffer A at pH 7 with a Pasteur pipette drop by drop along the tube wall until the precipitate was completely dissolved, and then centrifuged at 800 × g for 90 s. The resulting supernatant (approximately 18 mL) containing most of the crude plastids was evenly layered onto a discontinuous sucrose gradient (0.75, 0.92, 1.2, 1.50 M sucrose in 50 mM Tricine-NaOH, pH 6.8 supplemented with 1 mM DTT), with 2.5 mL of each gradient (top to bottom: 0.75, 0.92, 1.2 and 1.5 M), and subjected to centrifugation at 62,000 × g for 90 min in a Hitachi P40ST swing-bucket rotor. Plastid fractions, which were designated as bands 1, 2, 3, 4 from the top to the bottom of the gradient, were clearly separated at 0.75–0.92 M, 0.92–1.2 M and 1.2–1.5 M sucrose interfaces (Fig. 1). Each band was carefully collected by gentle aspiration with a Pasteur pipette and examined under an optical microscope (Cover-018; Olympus, Tokyo) at × 100 magnification (Fig. 1). The recovered band 2 and band 3 fractions (approximately 10–12 mL) were washed with one volume of buffer A at pH 6.8 and centrifuged at 6,000 × g for 10 min. The final pellets containing chloroplast or chromoplast fractions were collected and flash-frozen in liquid nitrogen and kept at − 80 °C for the following protein experiments (Fig. 1).

### Preparation of tissue sections and microscopy

To prepare frozen sections, the flavedo pieces (about 5 mm^3^) were fixed with 2% glutaraldehyde and 4% paraformaldehyde in a 20 mM sodium cacodylate buffer at pH 7.0 and 4 °C overnight. The fixed samples were embedded in super cryoembedding medium (Leica Microsystems K.K., Tokyo, Japan) within liquid N_2_. Thin cryosections were cut and transferred to glass slides, and then visualized by an Olympus BX61 microscope with a DP70 CCD camera (Olympus, Tokyo, Japan).

For light microscopy, the isolated plastids were carefully layered onto microscope slides and visualized using an Olympus BX61 microscope with a DP70 CCD camera (Olympus, Tokyo, Japan). Samples of flavedo tissues for transmission electron microscopy (TEM) analysis were prepared and observed as described by Zeng et al. [3].

### Confocal microscopy of isolated plastids

Florescence images of plastids isolated from three different stages were acquired using a confocal laser-scanning microscope (TCS SP8; Leica, Wetzlar, Germany). Confocal microscopy of isolated plastids was conducted as described by Egea et al. [24] with minor modifications. Samples of freshly isolated plastid fractions were mounted in vectashield. Red and green fluorescence represent the autofluorescence of chlorophyll and carotenoids, respectively. The samples were excited using an argon laser at the wavelength of 488 nm, and the emission fluorescence was recorded from 500 to 735 nm.

### BN-PAGE analysis

BN-PAGE analysis was performed as described by Kügler et al*.* [25] with some modifications.

#### a) Sample preparation

All reagents and materials were kept on ice throughout the experiment. The purified plastids were suspended in ice-cold resuspension buffer containing 25 mM BisTris-HCl (pH 7.0) and 20% glycerol, and then solubilized with 1% (w/v) dodecyl maltoside (β-DM) for 10 min. After centrifugation at 18,800 × g for 15 min at 4 °C, the supernatant was supplemented with loading buffer [5% (w/v) Serva Blue G, 500 mM 6-aminocaproic acid, 100 mM BisTris-HCl (pH 7.0) and 30% glycerol] at a detergent/dye ratio of 4:1 (w/w).

#### b) Blue-native PAGE

Samples were loaded directly onto a mini gel with 0.75 mm spacer consisting of a separating (linear 5–13.5% acrylamide) and a stacking gel (4% acrylamide). The gel compositions are outlined in Table 1. Each lane was loaded with 200 μg of total protein. A high molecular weight kit of standard proteins (HMW native protein marker kit, 17–0445-01, UKGE Healthcare, Amersham-Pharmacia) was used for molecular weight marker, and the migration of protein bands during electrophoresis was traced following the blue bands. Solutions for electrophoresis were: cathode buffer—50 mM Tricine/15 mM Bis–Tris, pH 7.0 (4 °C)/0.01% Serva Blue G, and anode buffer—50 mM Bis–Tris, pH 7.0 (4 °C). The electrophoresis was performed at 4 °C by using a Hoefer SE 600 vertical electrophoresis system (Hoefer SE600, GE Healthcare) equipped with a cooled circulating water system. Protein complexes were separated for about 2 h on a continuous BN gel with a constant voltage at 100 V (approximately 6 mA), and continued increasing the voltage by 50 V every 1 h for about 4–5 h. When the dye front reached the middle of the separation distance, the cathode buffer containing 0.01% Serva Blue G was replaced by colorless cathode buffer. The bands of protein complexes in the BN-gel were visualized directly and the image of the gel was obtained by a typhoon scanner (GE Typhoon FLA 7000). Calculation of the relative intensities of protein bands was performed with Quantity One^®^ 1-D software (Bio Rad).

**Table 1:** Gel compositions

	Sample gel	Gradient separation gel ^c^	
	4%	5%	13.5%
49.5% Acrylamide solution^a^	0.121 mL	0.212 mL	0.573 mL
3 × Gel buffer^b^	0.5 mL	0.7 mL	0.7 mL
75% Glycerin	0	0.14 mL	0.56 mL
Distilled water	0.86 mL	1 mL	0.22 mL
10% Ammonium persulfate	3 μL	2 μL	2 μL
N,N,N’, N’ Tetramethylethylendiamine	8 μL	6 μL	6 μL
Total volume	1.5 mL	2.06 mL	2.06 mL

^a^49.5% Acrylamide solution: 24 g acrylamide, 0.75 g bisacrylamide, 50 mL H_2_O. Store at RT

^b^3 × Gel buffer: 1.5 M 6-aminocaproic acid/0.15 M Bis–Tris, pH 7.0. Store at 4 °C

^c^Gradient separation gel: Prepare 5%, 13.5% gradient acrylamide mixtures, and use a peristaltic pump to divide them into 5–13.5% linear acrylamide solution

### Immunoblot analysis

The pericarp or plastid samples were initially resuspended in protein extraction buffer [50 mM Tris HCl, pH 7.5, 0.15 M NaCl, 1 mM Na_2_ EDTA, 1% NP-40, 10% glycerol, and 1 mM phenylmethylsulfonyl fluoride (PMSF)] and then sonicated on ice to completely dissolve pericarps or plastids. The lysate was centrifuged at 12,000 × g for 15 min at 4 °C. The supernatant was quantified using Bio-Rad Bradford assay kit. A total of 20 μg of proteins were loaded and separated by 15% sodium dodecyl sulfate polyacrylamide gel electrophoresis (SDS-PAGE). Two gels with identical protein loading were running at the same time, when the electrophoresis was finished, one gel was stained by Coomassie Blue for direct visualization, and the other was transferred to nitrocellulose membrane (Solarbio) using a wet transfer (Trans blot Electrophoretic-transfer Cell, Biorad) for 1 h at 100 V, then treated with blocking TBST buffer [20 mM TRIS–HCl, 150 mM NaCl, 0.05% (v/v) Tween-20] and subsequently incubated for 2 h with polyclonal antibodies diluted in TBST buffer as indicated in the manual. Specific antibodies were purchased from Agrisera and diluted appropriately, including polyclonal antibodies against translocon at the inner envelope membrane of chloroplasts 110 (Tic110; 110 kDa, 1:2000) and cytosolic UDP-glucose pyrophosphorylase (UDPase; 51.6 kDa, 1:2000), as well as photosynthetic antibodies against Lhca1–Lhca4, PsaA (1:5000) of PS I subunits and Lhcb1–Lhcb5, PsbA (1:5000) of PS II subunits. Antibody-bound proteins were detected using the Thermo Scientific^®^ Kit (SuperSignal West Pico Chem iluminescent Substrate) after incubation with goat anti-rabbit secondary antibodies [peroxidase-Conjugated immunoPure Goat Anti-Rabbit IgG (H + L)] at a 1:2000 dilution in TBST at room temperature for 1 h, and immune signals were imaged using a chemiluminescent gel imager (Amersham Imager 600).

### Protein extraction and two-dimensional electrophoresis (2-DE)

Plastids from fruit at different coloring stages were re-suspended with extraction buffer containing 1.5% SDS, 0.1 M Tris–HCl (pH 7.5), 2 mM EDTA-Na_2_, 20 mM DTT, and 2 mM PMSF (added before use). The extracts were centrifuged at 15,000 × g for 10 min, and the supernatant was subjected to phenol extraction as described by Isaacson et al. [26]. The protein pellet was dissolved in 2-DE rehydration buffer containing 7 M urea, 2 M thiourea, 2% (w/v) CHAPS, 20 mM DTT, and 0.5% (v/v) IPG buffer (Bio-Rad, USA). The protein concentration was determined using the Bio-Rad Bradford assay kit (Bio-Rad, USA).

For 2-DE, first dimension isoelectric focusing (IEF) separation was performed using 7 cm linear pH 4–7 IPG strips (Bio-Rad, USA). The strips were loaded with equal amounts of proteins (350 μg in 135 μl) and passively rehydrated for 12 h at 20 °C on PROTEAN IEF CELL system (Bio-Rad, USA). Then, the IEF voltage was set and held at 250 V for 30 min, 500 V for 30 min, 2000 V for 1.5 h, finally increasing to 4000 V for 3 h, and holding for 5 h. Focused strips were equilibrated in buffer I (0.1 M Tris–HCl, pH 8.8, 2% SDS, 6 M urea, 30% glycerol, 0.1 M DTT) and then in buffer II (same as Buffer I, but with 0.25 M iodoacetamide instead of DTT) for 15 min each. The strips were then transferred to 12% SDS-PAGE gels for second dimension electrophoresis by the Bio-Rad Mini-PROTEAN^®^ Tetra System (BioRad, USA). The program was set as follows: 80 V for 30 min, and then 180 V for 1 h for each strip. SDS-PAGE gel was stained with Coomassie brilliant blue (CBB) R350. Gel images were captured using the camera (Canon EOS 80D). Digital 2-DE images were processed and analyzed using PDQUEST 8.0 software (Bio-Rad), more information on parameter settings can be found in the Additional file.

## Results and discussion

### Ultrastructure of plastids from citrus fruit during chloroplast-to-chromoplast differentiation

**Fig. 2:**
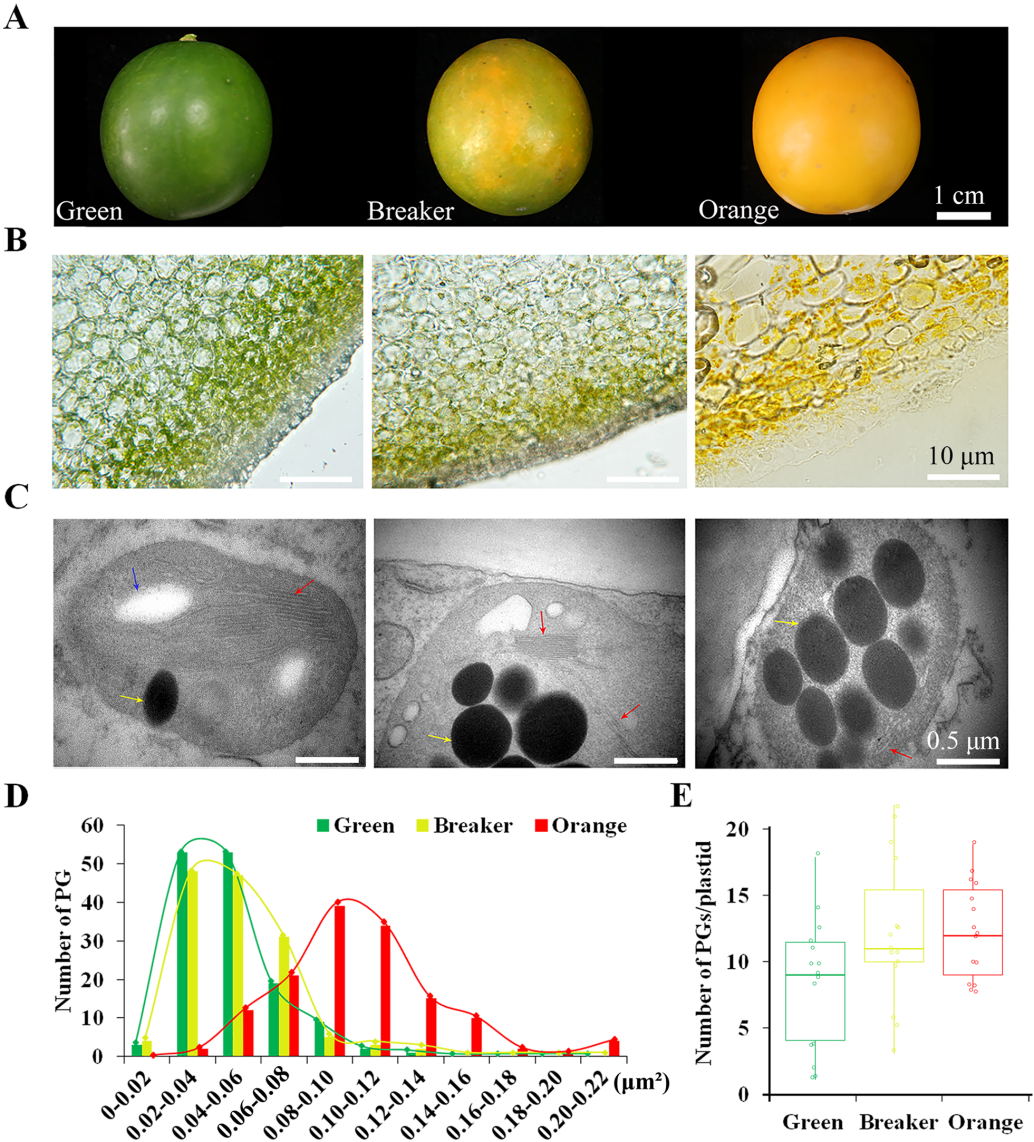
Plastids isolated from the outer peel of kumquat at three different coloring stages. **A** Representative photographs of kumquat fruit used for plastid isolation. From left to right: ‘Green’, ‘Breaker’ and ‘Orange’ represent the stage from immature green to mature orange phase. **B** Frozen sections of epicarp. **C** Transmission electron microscopic (TEM) images showing the ultrastructural features of plastids in the outer peel of kumquat. Red arrow shows thylakoid lamellar structure. Yellow arrow shows plastoglobule (PG). Blue arrow shows starch grains. **D** Statistical analysis of the size of PGs per plastid in flavedo at three different coloring stages from Fig. 2, C. The y-axis represents the number of PGs at a given size, which indicated on the x-axis. The statistics was performed for a total of 140 PGs from 15 plastids. **(E)** Statistical analysis of the average number of PGs per plastid in flavedo at different maturation stages. The statistics was calculated from 15 plastids selected randomly

To better understand the differentiation process of chromoplasts in citrus, the kumquat fruit at three representative coloring stages (green, breaker, orange) were collected and phenotypically compared (Fig. 2A). The results of the frozen sections from the outer peel showed pigment accumulation in the epidermal and pericarp cells along with fruit development. At the early stage of fruit development, most of pericarp cells were occupied by intact and unaggregated green chloroplasts. In contrast, yellow chromoplasts were found to aggregate into clumps at the orange color stage of the fruit (Fig. 2B). Such aggregation of chromoplasts along with fruit development may imply that plastid membranes are partially degraded or disrupted at the fully ripening stage. The ultrastructure of plastids in green fruit peel still exhibited typical chloroplast structural features with lamellar and granal structures and starch granules (Fig. 2C). At the breaker stage, the plastids in the peel cells were mostly spherical chromoplasts, and their thylakoid systems were disintegrated and replaced by a few non-chlorophyllous single thylakoids, with the accumulation of large osmiophilic plastoglobules (Fig. 2C). At the orange color stage, the typical structure of developed chromoplasts was observed in pericarp cells, with some residual thylakoid membranes (Fig. 2C). These changes in plastid structure suggested a decrease in chlorophyll biosynthesis and an increase in carotenoid biosynthesis during the coloring of citrus fruit. These observations were confirmed by the increase in size and number of plastoglobules in fully mature fruit compared with those at early stages (Fig. 2D, E). Plastoglobules participate in lipid remodeling as functional micro-structures coupled to thylakoid membranes [27]. Both thylakoid disassembly and plastoglobule enlargement suggested the remodeling of plastid membranes during differentiation of chloroplasts into chromoplasts in citrus fruit.

### Isolation of plastids and purity evaluation

**Fig. 3:**
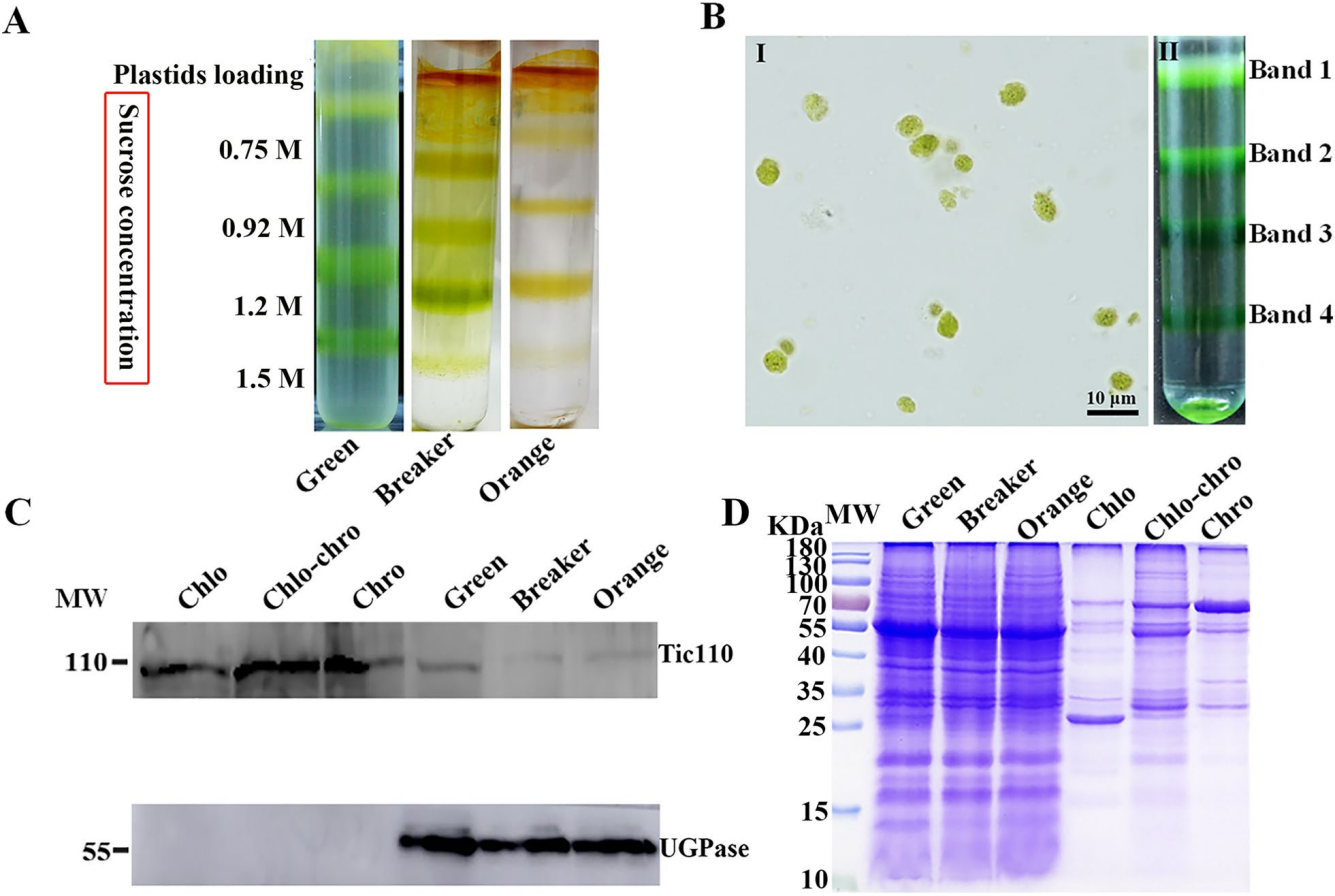
Plastid isolation and purity assessment. **A** Separation of plastids on a discontinuous sucrose gradient (0.75, 0.92, 1.20, 1.50 M) at three different coloring stages. **B** Light microscopy of purified chloroplasts from band 2 (I), plastid fractions were designated as bands 1, 2, 3, 4 from the top to the bottom of the sucrose gradient (II). **C** Assessment on the purity of isolated plastids using immunoblots. Purified plastids (band 2) were detected using antibodies for Tic110 (translocon at the inner envelope membrane of chloroplasts) and cytosolic UGPase. Each lane was loaded with 20 µg of total protein. The positions of the molecular markers are indicated on the left. Chlo, chloroplast; Chlo-Chro, intermediate plastids between chloroplasts and chromoplasts; Chro, chromoplast. **D** Coomassie blue protein profiles of purified plastids (band 2) from kumquat peel as compared with total kumquat peel proteins

Plastids were extracted from fruit at different coloring stages by sucrose density gradient centrifugation (Fig. 3A). The collected pellet containing both chloroplasts and chromoplasts at each developmental stage was separated into four bands on a discontinuous gradient comprising 0.75, 0.92, 1.20, and 1.50 M sucrose (Fig. 3A). The upward trend of the band for plastid distribution demonstrated that chromoplasts were of a lower density relative to chloroplasts in citrus fruit. The plastids localized in band 2 and band 3 were collected for subsequent study in consideration of possible contamination of band 1 and band 4 by either cell debris or other organelles. The chloroplasts were characterized by abundant green sphere with membrane structures, and their sizes ranged from 2 to 6 μm as measured under light microscopy (Fig. 3B). Sucrose gradient centrifugation was used to isolate plastids from different citrus cultivars, including ‘Rong An’ kumquat and ‘Hong Anliu’ sweet orange Additional file 1: Fig. S1). It was found that ‘Hua Pi’ kumquat produced the best isolation among all tested varieties, which may be related to its low oil and acid properties, while the integrity and purity were slightly lower in the plastids isolated from ‘Rong An’ kumquat and ‘Hong Anliu’ sweet orange (Additional file 1: Fig. S1).

The purity of the plastid-enriched fractions was further assessed by western blot analysis with antibodies against Tic110 and UDPase. As shown in Fig. 3C, higher level of Tic110 was detected in the protein extracted from the purified plastid fragment relative to that from the exocarp, indicating an enrichment of plastids in the purified plastid fragment. Conversely, cytosolic UGPase was detected in the total proteins extracted from the kumquat peel, but not in purified plastid proteins (Fig. 3C). These results indicate that the isolated plastids were of high purity, and can be used for subsequent analysis. It should be noted that protein bands on SDS-PAGE gel were reduced remarkably in purified plastids than in exocarp crude extracts (Fig. 3D), suggesting that most non-plastid localized proteins were likely removed using the sucrose gradient.

### Pigment assessment during the transition of chloroplasts to chromoplasts

**Fig. 4:**
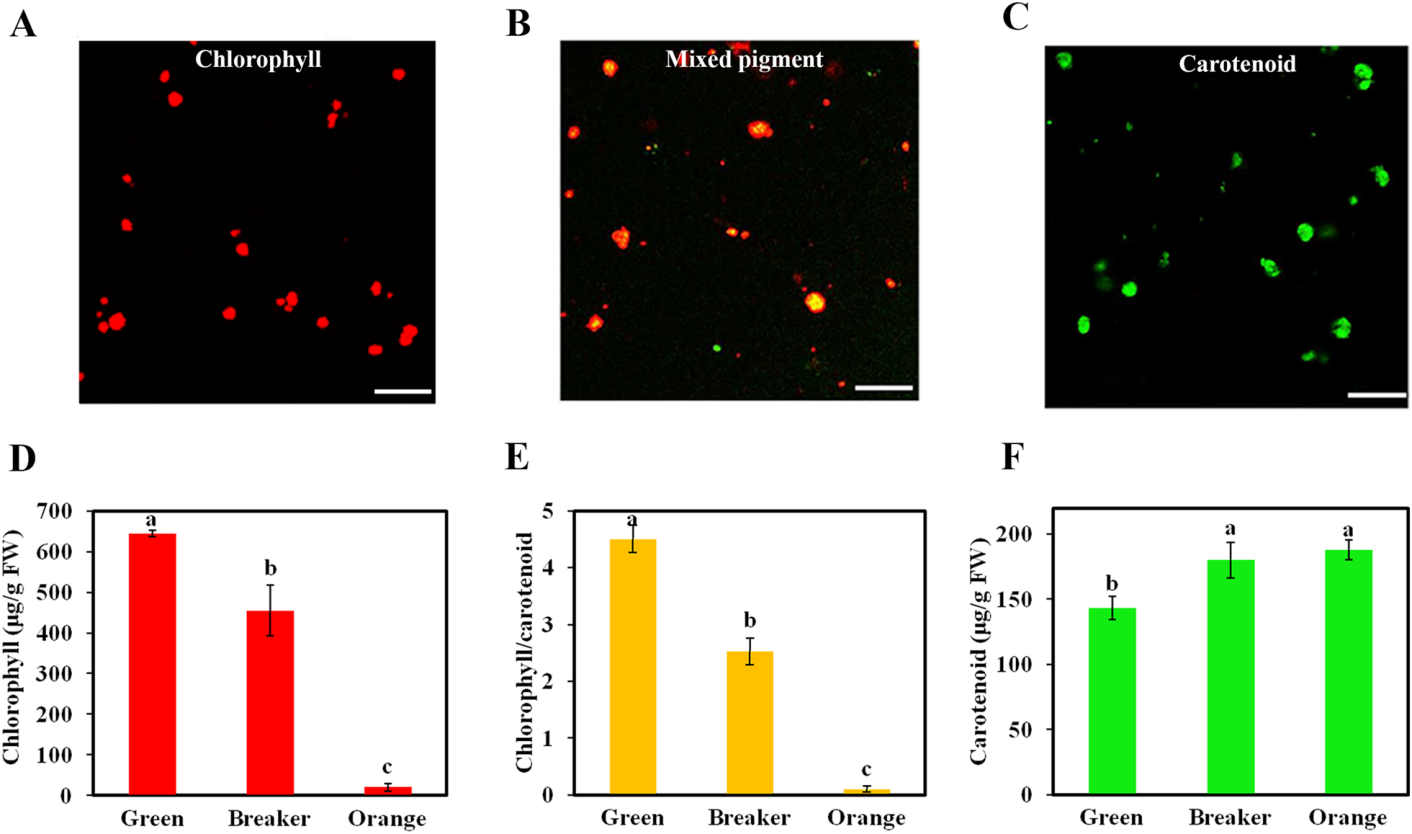
Confocal images of purified plastids and analysis of pigment content. Confocal images of chloroplasts **A**, intermediate plastids **(B)** and mature chromoplasts **(C)** suspensions isolated from green, breaker and fully mature pericarp of kumquat, respectively. Images are overlays of chlorophyll autofluorescence emitted at 650–700 nm (red) and carotenoid autofluorescence emitted at 500–600 nm (green), respectively. The extracts mainly containing chlorophyll appear red, those only containing carotenoids appear green, and those containing both chlorophyll and carotenoids appear yellow. Bar, 25 μm. The excitation wavelength was 488 nm. The peaks of fluorescence emission around 520–550 nm and 680 nm correspond to carotenoids and chlorophylls in citrus fruit, respectively. Measurement of chlorophyll concentration (**D**), chlorophyll/carotenoid ratio (**E**) and carotenoid concentration (**F**) in kumquat fruit at green, breaker and orange color stages, respectively. The pigment concentration is recorded with the unit of μg g ^−1^ fresh weight

Confocal observation of isolated plastids can provide more insights into the changes in pigment accumulation at cellular level along with fruit development. As shown in Fig. 4, the green fruit was rich in chloroplasts, with emission of chlorophyll autofluorescence within the range of 650–700 nm, while the autofluorescence of carotenoids was not detected (Fig. 4A). Plastids isolated from the fruit at the breaker stage exhibited mixed autofluorescence signal from both chlorophylls and carotenoids, resulting in a yellow color in the overlay picture (Fig. 4B), suggesting that this fraction stands for an intermediate stage in the differentiation of chloroplasts into chromoplasts. At the late maturation stage, the fluorescence was mostly detected for carotenoids (emission spectrum at around 550 nm) in the suspension of chromoplasts, and little fluorescence was detected for chlorophyll (Fig. 4C). Changes in the chlorophyll/carotenoid ratio reflect variations in the composition and ratio of chloroplasts and chromoplasts in higher plants. Here, it was found that the chlorophyll/carotenoid ratio decreased sharply from 4.5 in green chloroplasts to below 0.5 in fully differentiated chromoplasts (Fig. 4E), which could be ascribed to the decline of chlorophyll (Fig. 4D) and increase of carotenoids (Fig. 4F) during the transition of chloroplasts to chromoplasts, and is consistent with the observation of pigment fluorescence in individual plastids (Fig. 4).

### Profiles of macromolecular protein complexes in citrus plastids by BN-PAGE analysis

**Fig. 5:**
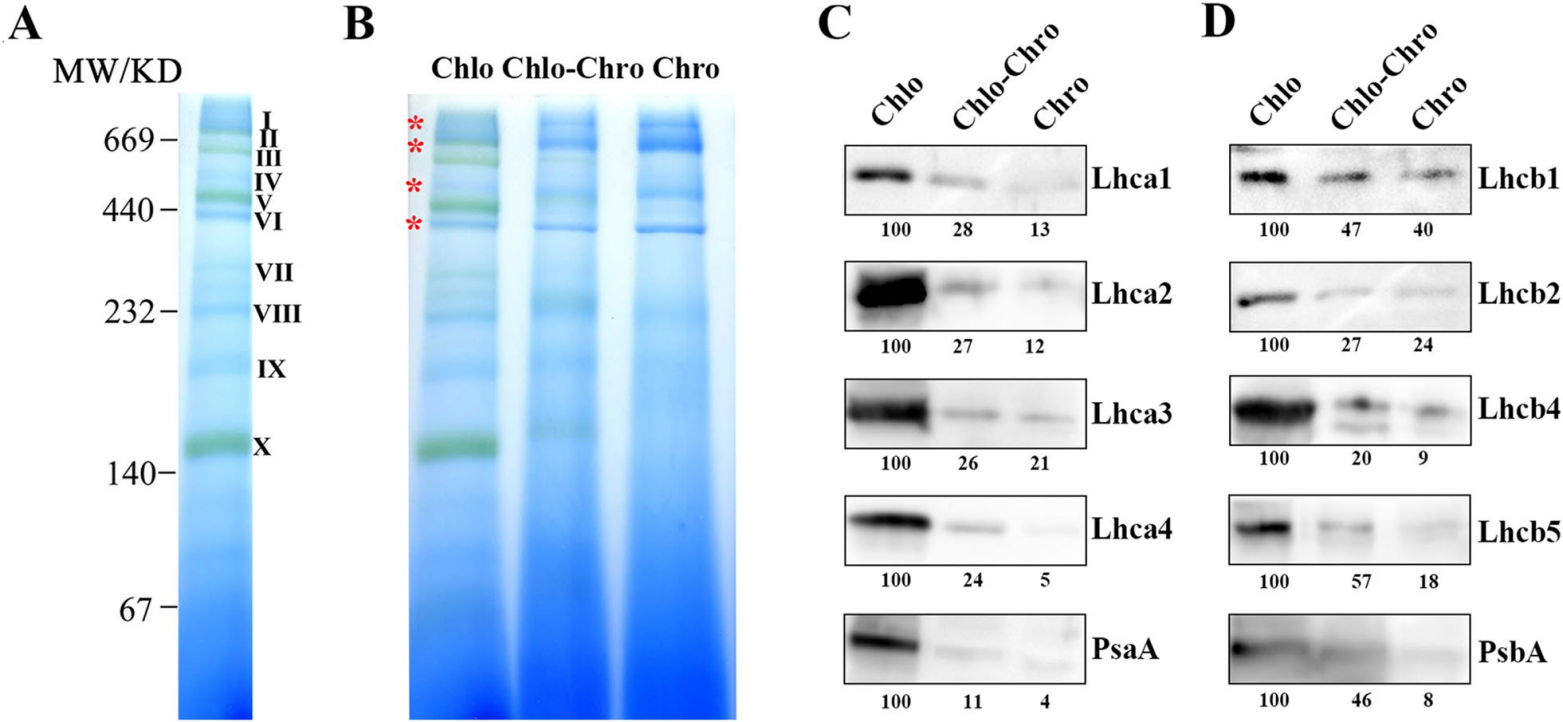
Blue-native PAGE profile and immunoblot with antibodies against photosynthesis-related proteins from isolated plastids at green, breaker and orange color stages. **A** Blue-native PAGE profile of chloroplast membrane protein complexes at green stage. Molecular mass markers are indicated on the left of the figure. **B** Separation of membrane protein complexes from chloroplasts (left lane), chlo-chromoplasts (middle lane) and chromoplasts (right lane) by BN-gel electrophoresis. Red asterisks indicate the bands present in all three periods. Each lane of BN-gel was loaded with 200 μg of protein. Immunoblotting analysis showing changes in the relative amounts of protein subunits in PS I complexes **(C)** and PS II complexes **(D**). Each lane of immunoblotting analysis was loaded with 20 μg of protein

In the present study, BN-PAGE analysis was introduced to investigate the profiles of macromolecular protein complexes during plastid differentiation in citrus fruit with purified plastids from the peel at representative coloring stages. Plastid protein complexes were successfully separated from kumquat fruit by using BN-PAGE technique, and the separated bands were directly visible. Ten major bands of protein complexes were obtained for citrus chloroplasts as shown in Fig. 5A. According to previous reports [28–30], and combined with high molecular weight calibration, the possible complexes from each band were interpretated and shown in Table 2, including supercomplexes (band I), PS II-LHCII MC (band II), PS II-LHCII SC (band III), F_0_F_1_-ATPase (band IV), PS I, PS II-D (band V), F1-ATPase (band VI), PS II Core + Cytb_6_f (band VII), PS II-M (band VIII), LHCII assembly (band IX) and trimeric LHCII (band X). The BN-PAGE analysis demonstrated that protein complexes of PS II-LHCII SC (band III), PS I, PS II-D (band V), PS II Core + Cytb_6_f (band VII) and trimeric LHCII (band X) significantly decreased, while supercomplexes (band I), PS II-LHCII MC (band II), F_0_F_1_-ATPase (band IV) and F1-ATPase (band VI) were present in all three periods (Fig. 5B and Additional file 1: Fig. S2), suggesting the degradation of chloroplast membrane protein complexes in an orderly and asynchronous manner. The appearance of green or yellow bands indicated the binding of a large amount of pigment to the protein. Light-harvesting chlorophyll-binding proteins (LHCPs) are usually the most abundant membrane proteins in chloroplasts, and it could be intuitively observed that they were gradually degraded during the progress of fruit coloring (Fig. 5B). With the loss of chlorophyll, we found that the chlorophyll-protein complexes also underwent obvious hydrolysis, indicating that chlorophyll is an essential stabilizer of chlorophyll-protein complexes, which is consistent with previous reports [31, 32]. In addition, previous studies have shown that there is a defect in chlorophyll degradation when the degradation of light-harvesting complex II (LHCII) is inhibited [33, 34], suggesting that the hydrolysis of LHCPs and chlorophyll degradation are a prerequisite for each other.

**Table 2:** Apparent MW of predicted chloroplast membrane complexes from kumquat (*F. crassifolia* Swingle)

Band	Complexes	MW(kDa)
I	Supercomplexes	709
II	PSII-LHCII MC	678
III	PSII-LHCII SC	647
IV	F_0_F_1_-ATPase	548
V	PSI,PSII-D	–
VI	F1- ATPase	327
VII	PSII Core+Cytb/6f	309
VIII	PSII-M	231
IX	LHCII assembly	–
X	LHCII-T	165

*MC* megacomplexes, *SC* supercomplexes, *M* monomers, *D* dimmers, *T* trimers

Compared with other methods for the separation of protein complexes, including size exclusion chromatography, sucrose density ultracentrifugation, or sample filtration through membranes with distinct molecular weight cutoffs, BN-PAGE is superior in terms of resolution and the isolation of native protein complexes with high molecular weights [35]. Although citrus is a relatively complexed species with various and abundant secondary metabolites, BN-PAGE could still produce high resolution bands for the separation of pigment-protein complexes from fruit (Fig. 5A, B). The limitation is that protein complexes of the same size may migrate together in a single band, with the migration behavior depending on the size, shape and even post-translational modification of the complex [36, 37], which may lead to prediction errors of the protein complexes. Therefore, it is necessary to use complementary techniques to verify the observed interactions. For example, combining BN-PAGE with two different denaturing gel electrophoresis steps (Tris-Tricine-urea and SDS-PAGE) is capable of disassembling the protein components, whose identity can be verified by mass spectrometry [38, 39]. It is even possible to distinguish proteins that only differ in post-translational modification. In addition, it is suitable to identify the interaction between the protein and RNA components with subsequent application of northern blotting [35]. Thus, the BN-PAGE system may be a promising tool for studying protein–protein or protein-RNA interaction in fruit.

### Semi-quantification of protein changes during the plastid transition of citrus fruit

In order to further elucidate the changes in the protein composition of chloroplasts, we analyzed the abundance of subunits of thylakoid membrane complexes by western blotting. The imunoblotting results revealed that the four light-harvesting chlorophyll a/b-binding proteins (LHC) of PS I (Lhca1-4) and the PS II antenna proteins Lhcb1-2 as well as Lhcb4-5 were preferably accumulated in chloroplasts, and declined sharply in parallel along with fruit coloring progress (Fig. 5C, D). The PsaA (a core protein of PS I) and PsbA (D1 protein of PS II) proteins were also accumulated in chloroplasts, but their amounts almost dropped to zero in fully mature chromoplasts (Fig. 5C, D). A sharp and continuous decrease in thylakoidal proteins and chlorophyll content implied the structural differentiation to chromoplasts [40]. The decline in the relative level of light-harvesting complex of PS I was more significant than that of PS II, which is in line with the previously reported results on the photosynthetic changes of flag leaves during senescence stage in super high-yield hybrid rice [41]. Furthermore, the degradation rate of each chlorophyll-protein complex was different, and the stability of chlorophyll-protein complexes during the transition from chloroplasts to chromoplasts approximately followed the order of LHCII > PS II core > LHC I > PS I core in citrus fruit.

We have also introduced the 2D-gel electrophoresis method to analyze the plastid-associated changes in proteome profiles during the coloring process of citrus fruit. The 2D-PAGE of the green stage of chloroplasts showed 150 protein spots, while 156 and 108 protein spots were detected in the intermediates and chromoplasts, respectively (Additional file 1: Fig. S3). Among the plastids at three stages, the intermediate plastids displayed the largest number of protein spots, and showed a high matching rate with chloroplast and chromoplast proteome, suggesting that chloroplasts differentiated to chromoplasts during this period. On one hand, three types of plastids showed similar distribution patterns of protein dots (Additional file 1: Fig. S3), implying that the plastid proteomes are conserved. On the other hand, many proteins exhibited dramatic changes in abundance (red arrow), suggesting certain specificity of the proteins among plastids. These results are in line with those reported in previous studies on the proteomic analysis of plastids in sweet orange fruit and tomato [18, 40].

## Conclusions

**Fig. 6:**
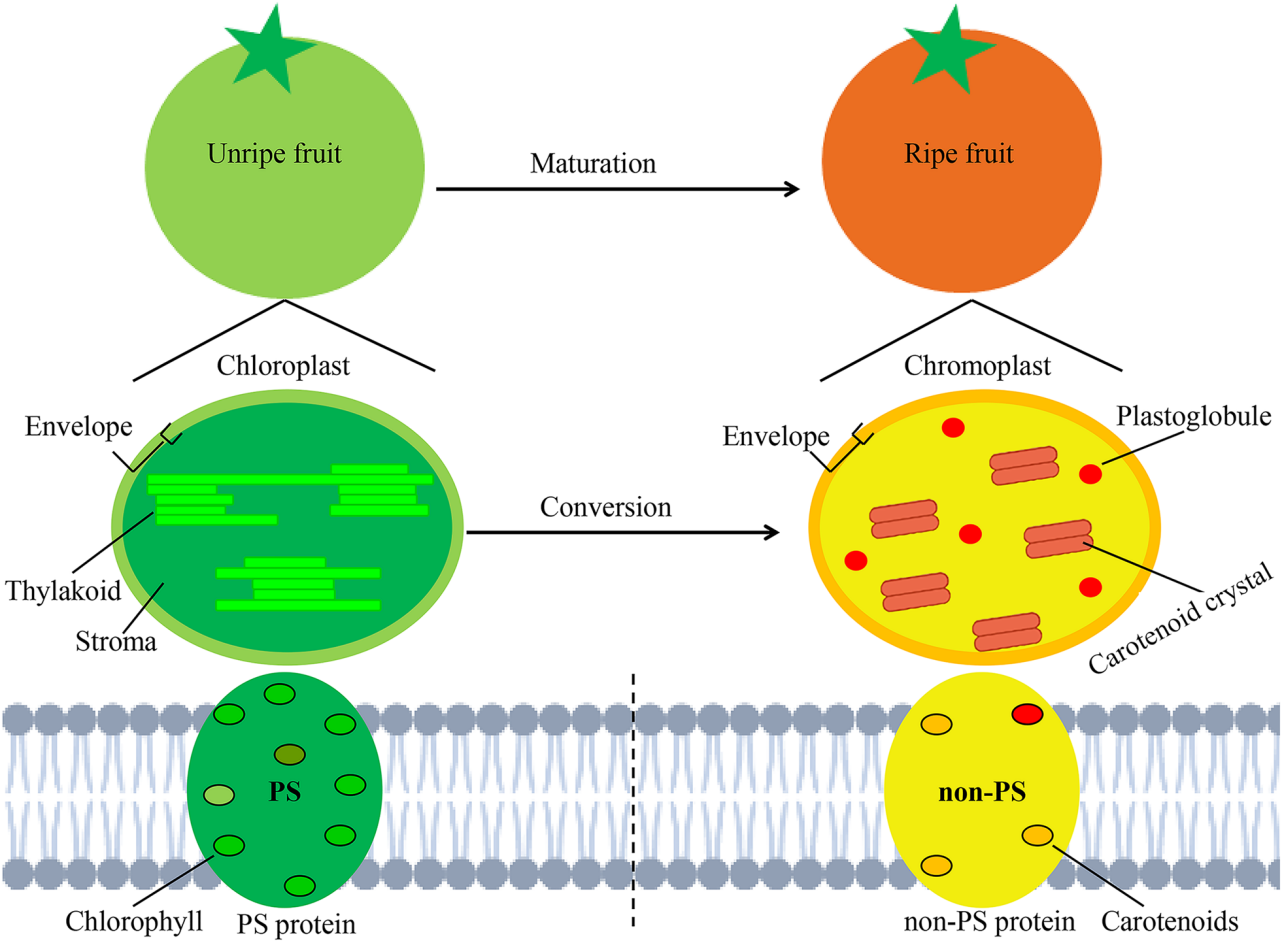
Model of the chloroplast-to-chromoplast differentiation process during fruit ripening, accompanied by disruption of the chlorophyll-photosynthetic (PS) protein complexes and extensive synthesis of the carotenoid-non-PS protein complexes

Biogenesis of chromoplasts, especially the transition of chloroplasts to chromoplasts, is an interesting topic in the studies of fruit ripening, because it is not only essential for vital cellular processes of plants, but also important to human consumption for the health and aesthetic benefits. Many physiologists specialized in plastid studies have explored the fruit physiology by characterizing the differentiation of plastids during fruit ripening [42]. In this work, the model of the chloroplast-to-chromoplast transition during citrus fruit maturation is shown in Fig. 6, in which remodeling of the plastid membranes system and formation of carotenoid storage structures are represented. We observed the changes in the morphology and abundance of plastids during the development of citrus fruit. The isolated plastids were used to monitor the changes in carotenoids and chlorophyll by laser scanning confocal microscopy, and extract the macromolecular protein complexes under non-denaturing conditions, as well as analyze the protein distribution patterns by 2D electrophoresis under denaturing conditions. The important finding is the pigment-protein complex profiles in fruit, and we also found that there is a dramatic decrease in the abundance of chlorophyll-protein complexes and photosynthesis-related proteins during fruit ripening, which is accompanied by the loss of chlorophyll due to the absence of thylakoid components and accumulation of carotenoids. Taken together, we have improved the method for isolating plastids from citrus fruit by using ‘Hua Pi’ kumquats, and successfully introduced BN-PAGE technique to separate pigment-protein complexes under natural conditions from fruit for the first time. The results reveal the differentiation of chloroplasts to chromoplasts in citrus fruit from multiple perspectives. Further protein identification combined with all these findings and physiological data may provide more in-depth insights into the metabolic and physiological status of fruit.

## Supplementary Information

**Additional file 1: Figure S1**. Separation of plastids from exocarp of ‘Rong An’ kumquat and ‘Hong Anliu’ sweet orange on a discontinuous sucrose gradient, and light microscopy of isolated plastids from band 3. **Figure S2.** Normalized volume of ten protein complexes from BN-PAGE, MCPs, multiple protein complexes. **Figure S3.** 2D gel electrophoresis of plastid proteins in citrus fruit.

## Data Availability

The datasets used and/or analyzed during the current study are available from the corresponding author on reasonable request.
