# Supplementary material for: Combining BN-PAGE and microscopy techniques to investigate pigment-protein complexes and plastid transitions in citrus fruit
Source: Plant Methods. 2022 Nov 19;18:124. doi: 10.1186/s13007-022-00956-1 (PMC9675244; doi:10.1186/s13007-022-00956-1)
Supplement: Supplementary file 1 — Additional file 1: Figure S1. Separation of plastids from exocarp of ‘Rong An’ kumquat and ‘Hong Anliu’ sweet orange on a discontinuous sucrose gradient, and light microscopy of isolated plastids from band 3. Figure S2. Normalized volume of ten protein complexes from BN-PAGE, MCPs, multiple protein complexes. Figure S3. 2D gel electrophoresis of plastid proteins in citrus fruit. [file 13007_2022_956_MOESM1_ESM.doc]

Supplementary figures for

**Combining BN-PAGE and microscopy techniques to investigate pigment-protein complexes and plastid transitions in citrus fruits**

Jinli Gong^1,2,3^, Hang Zhang^2,3^, Yunliu Zeng^2,3^, Yunjiang Cheng^2,3^, Xuepeng Sun^1*^, Pengwei Wang^2,3*^

^1^Collaborative Innovation Center for Efficient and Green Production of Agriculture in Mountainous Areas of Zhejiang Province, College of Horticulture Science, Zhejiang A&F University, Hangzhou, Zhejiang 311300, China.

^2^Key Laboratory of Horticultural Plant Biology (Ministry of Education), College of Horticulture and Forestry Science, Huazhong Agricultural University, Wuhan, Hubei 430070, China.

^3^National R&D Centre for Citrus Preservation, Huazhong Agricultural University, Wuhan, Hubei 430070, China.

*For correspondence. E-mail address: xs57@zafu.edu.cn；[wangpengwei@mail.hzau.edu.cn](mailto:wangpengwei@mail.hzau.edu.cn)


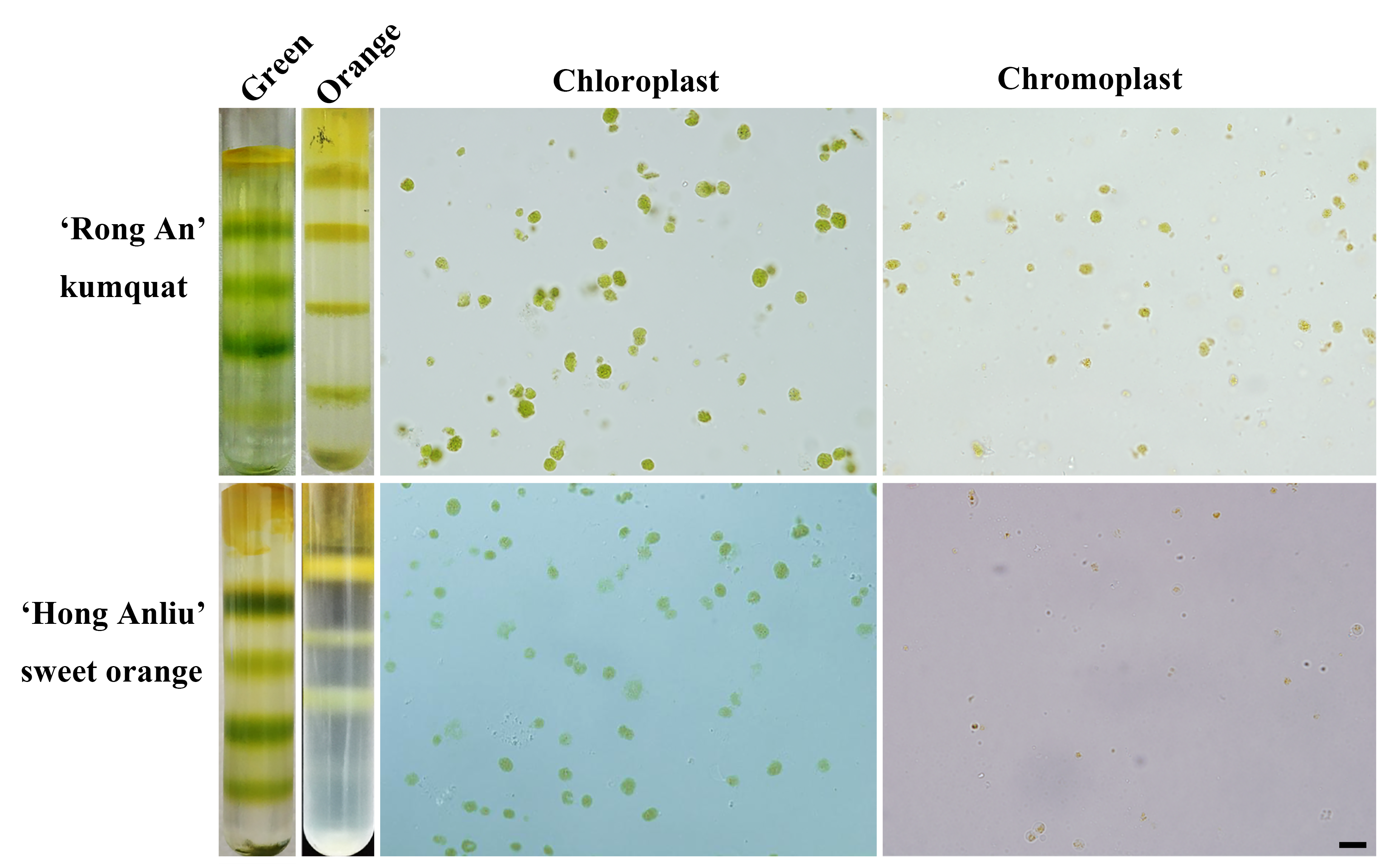


**Figure S1.** Separation of plastids from exocarp of ‘Rong An’ kumquat and ‘Hong Anliu’ sweet orange on a discontinuous sucrose gradient, and light microscopy of isolated plastids from band 3. Bar=10 μm.


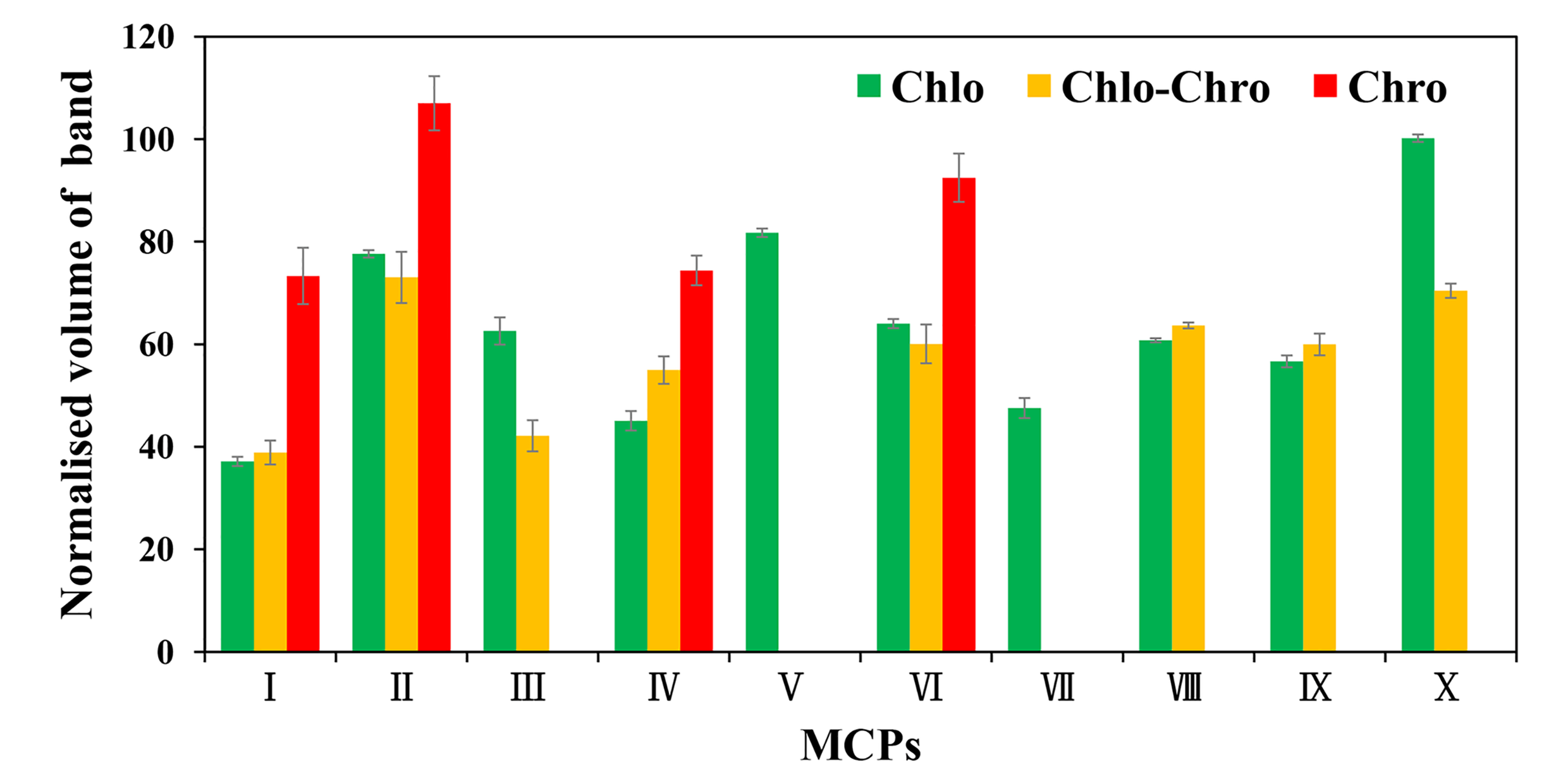


**Figure S2.** Normalized volume of ten protein complexes from BN-PAGE, MCPs, multiple protein complexes.

**
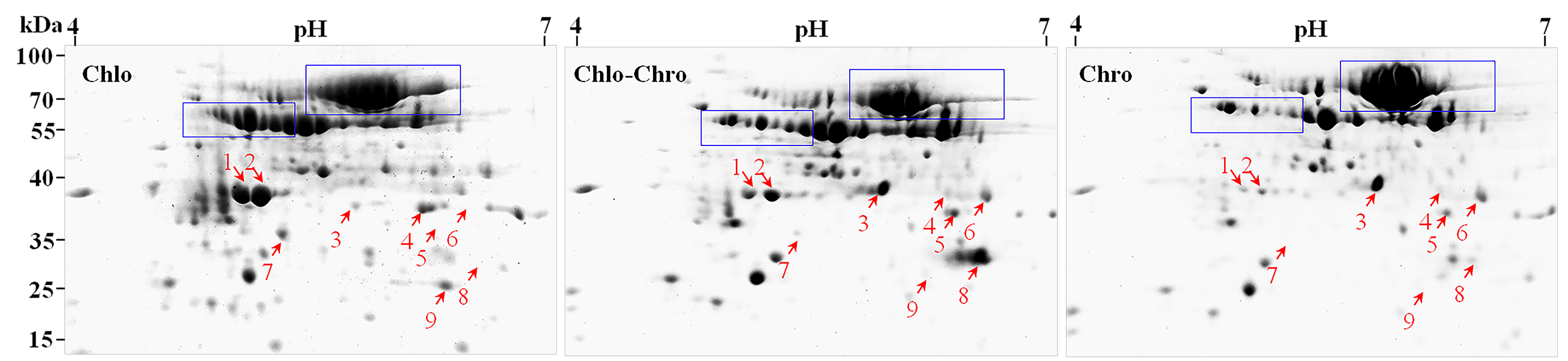
Figure S3. 2D gel electrophoresis of plastid proteins in citrus fruit.** Plastid proteins (350 μg) were resolved by IEF using 7 cm pH 4–7 IPG dry strip. Secondary SDS-PAGE was carried out on a 12% resolving gel. Spots of relatively abundant proteins in plastids at different stages with at least two-fold change in abundance are indicated with red arrows. Blue boxes indicate the regions with differential subunit forms. Spot volumes were determined using the PDQuest software.
